# Infection status outcome, machine learning method and virus type interact to affect the optimised prediction of hepatitis virus immunoassay results from routine pathology laboratory assays in unbalanced data

**DOI:** 10.1186/1471-2105-14-206

**Published:** 2013-06-25

**Authors:** Alice M Richardson, Brett A Lidbury

**Affiliations:** 1 Faculty of Education, Science, Technology & Mathematics, University of Canberra ACT 2601, Canberra, Australia; 2 Department of Genome Biology, The John Curtin School of Medical Research, The Australian National University ACT 0200, Canberra, Australia

## Abstract

**Background:**

Advanced data mining techniques such as decision trees have been successfully used to predict a variety of outcomes in complex medical environments. Furthermore, previous research has shown that combining the results of a set of individually trained trees into an ensemble-based classifier can improve overall classification accuracy. This paper investigates the effect of data pre-processing, the use of ensembles constructed by bagging, and a simple majority vote to combine classification predictions from routine pathology laboratory data, particularly to overcome a large imbalance of negative Hepatitis B virus (HBV) and Hepatitis C virus (HCV) cases versus HBV or HCV immunoassay positive cases. These methods were illustrated using a never before analysed data set from ACT Pathology (Canberra, Australia) relating to HBV and HCV patients.

**Results:**

It was easier to predict immunoassay positive cases than negative cases of HBV or HCV. While applying an ensemble-based approach rather than a single classifier had a small positive effect on the accuracy rate, this also varied depending on the virus under analysis. Finally, scaling data before prediction also has a small positive effect on the accuracy rate for this dataset. A graphical analysis of the distribution of accuracy rates across ensembles supports these findings.

**Conclusions:**

Laboratories looking to include machine learning as part of their decision support processes need to be aware that the infection outcome, the machine learning method used and the virus type interact to affect the enhanced laboratory diagnosis of hepatitis virus infection, as determined by primary immunoassay data in concert with multiple routine pathology laboratory variables. This awareness will lead to the informed use of existing machine learning methods, thus improving the quality of laboratory diagnosis via informatics analyses.

## Background

Data mining approaches have found applications in many knowledge discovery domains, including biological research and clinical medicine [1-7]. Within data mining developments over the past twenty years, decision tree (recursive partitioning) learning models have received considerable attention. Decision trees are popular for several reasons, for example, the capacity to model complex relationships with logical rules. As Negevitzky (2002) [3] points out, they are also simple, easy to understand, and can be constructed relatively quickly.

In general, learning models are multi-stage decision processes that start with an initial set of datasets, which consists of various observations or cases for which a known class label has been assigned. In each dataset, segmentation algorithms look at known facts stored in a knowledge base and perform a series of tests in a specific order. At each stage of this process a decision is made and some records are separated into subsets with greater purity in terms of the class membership. This process usually continues until no more rules can be found or some stopping criterion is fulfilled. A decision tree model is a specific example of a learning model, and is represented by a tree structure. The tree structure consists of nodes, non-leaf nodes and branches. The non-leaf nodes represent the attributes and leaf nodes represent the values of the attribute to be classified.

Such learning models were applied to abundant diagnostic pathology laboratory data resulting from the testing of patients suspected of infection by either hepatitis B virus (HBV) or hepatitis C virus (HCV). Such data has not been extensively mined for patterns to advance predictions for laboratory diagnoses. Pathology data presents special challenges for investigators, including data imbalance for particular responses or predictors, and high individual patient data variation that makes both pattern recognition and rule detection difficult. Pathology data is similar worldwide, and therefore efficient analysis of such data is of wide interest to the clinical professions for enhanced laboratory diagnoses.

The immunoassay marker examined for HBV infection was hepatitis B surface antigen (HBSA), and for HCV a polyclonal anti-HCV antibody response (HepC). As well as the specific immunoassay data, case-associated routine diagnostic pathology variables were included in the pattern recognition analyses (Table 1). Both HBV and HCV are of widespread health significance as leading causes of liver disease worldwide [8-10], and responses to HBV or HCV infection as reflected by routine pathology variables, such as liver function test enzyme profiles (e.g. alanine amino transferase: ALT), are crucial to diagnosis and treatment monitoring. We demonstrate that the choice of key characteristics of data and decision tree algorithms can improve the sensitivity and specificity of diagnostic laboratory decision-making (beyond the sensitivity and specificity of the assays themselves), encouraging other pathology laboratories to conduct similar experiments on appropriate data.

**Table 1:** Description of response and explanatory variables subjected to decision tree analyses

**Variable abbreviation**	**Description & definition**	**Measurement units**
	**Response variable**	
HBSA	Hepatitis B Surface Antigen (marker of HBV infection)	Positive (1) or
HepC	Patient antibody to HCV, indicating contact with virus *(Both HBSA and HepC detected by immunoassay)*	Negative (0)
	**Explanatory variable**	
Age	Patient (case) Age	Years
Sex	Gender: 1 = F, 2 = M	M or F
ALT	Alanine aminotransferase (An intracellular enzyme released in after liver & other tissue cell damage)	U/L
GGT	Gamma-glutamyltranspeptidase (An intracellular enzyme also relevant to liver damage)	U/L
Hb	Haemoglobin	g/L
Hct	Haematocrit (formerly known as “packed cell volume”)	%
Mch	Mean corpuscular haemoglobin	pg/RBC
MCHC	Mean corpuscular haemoglobin concentration	g/L
MCV	Mean corpuscular volume	fL
Plt	Platelets (blood clotting)	x 10^9^/L
WCC	White cell count	x 10^9^/L
RCC	Red cell count	x 10^12^/L
RDW	Red cell distribution width	%
Neut	Neutrophil. White blood cell, elevated by bacterial infection and early viral infection	x 10^9^/L
Lymph	Lymphocyte. White blood cell, elevated by viral infection and some cancers	x 10^9^/L
Mono	Monocyte. White blood cell, elevated by infection, inflammation, some cancers.	x 10^9^/L
Eos	Eosinophil. White blood cell, elevated by allergy and parasite infection	x 10^9^/L
Bas	Basophils. White blood cell, elevated in hypersensitivity reactions.	x 10^9^/L

*U/L* Units per litre, *g/L* grams/Litre, *pg* picograms, *fL* femtolitres.

In this study we describe an empirical investigation of immunoassay results (HBV or HCV) and associated routine pathology data (Table 1), which featured significantly more negative than positive HBV or HCV cases, by constructing single decision trees and ensembles [11-13], and using different data pre-processing techniques on the aggregated pathology data. The aim of the study was to use the resulting trees for the enhanced laboratory diagnosis of hepatitis virus infection, by exploiting the range of multi-variable pathology laboratory data associated with direct virus immunoassay testing. To achieve this aim we interrogated a data set of 18625 records from 1997 – 2007 made available by ACT Pathology at The Canberra Hospital, ACT Australia.

## Results

### Single decision tree

**Figure 1:**
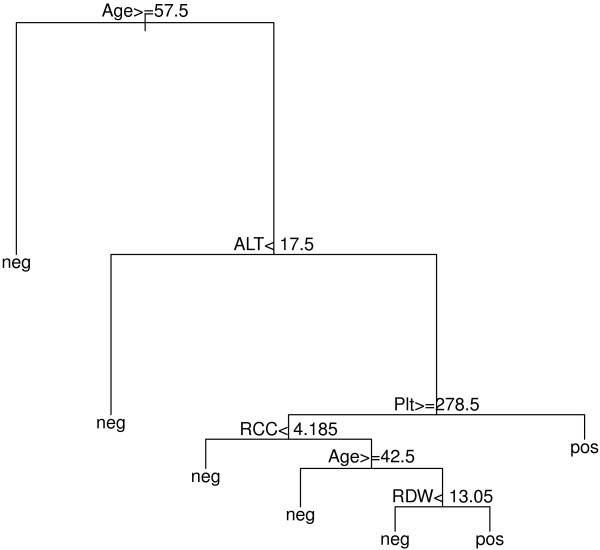
**A representative decision tree.** From the matched single analysis featuring popular explanatory variables associated with the HBSA response variable.

Table 2 shows that for negative HBSA and negative HepC predictions based on a single tree, derived from routine pathology test explanatory variables (Table 1), the “basic single” approach was extremely effective (sensitivity = 98-99%). This is associated with the large sample set available for patients who tested negative to HBV or HCV. For positive HBSA and HepC cases, where patient sample size was limited (n = 212 and n = 641 respectively), the basic single approach was very poor at predicting a positive HBSA (specificity < 5.0%), but improved for predicting a positive HepC result, most likely a result of the 3-fold larger sample size. (Specificity represents the accuracy of negative case detection, while sensitivity represents the accuracy of positive case detection: see Methods). For both positive HBSA and HepC cases, the maximum accuracy rates of approximately 65% were achieved though applying the “matched single” approach of data pre-processing. For positive cases, matched single pre-processing was significantly superior to the other data selection option tested, bootstrap single. Interestingly, prediction accuracy rates were reduced significantly for HBSA and HepC negative cases after bootstrap single pre-processing, with matched single processing further reducing the accuracy rates compared to the other methods. A representative decision tree from the above described single tree analysis is shown in Figure 1.

**Table 2:** Specificity and sensitivity (%) of HBV and HCV immunoassay outcome prediction after single decision tree analysis

**Measure**	**Basic single**	**Bootstrap single**	**Matched single**
HBSA specificity	98.38	89.57	45.65
HBSA sensitivity	4.6	16.92	64.62
HepC specificity	99.17	83.03	65.88
HepC sensitivity	32.35	35.29	65.89

See Software Methods for sensitivity and specificity calculations, and Phase 1 Methods for descriptions of the decision tree analyses.

### Ensemble analysis

**Figure 2:**
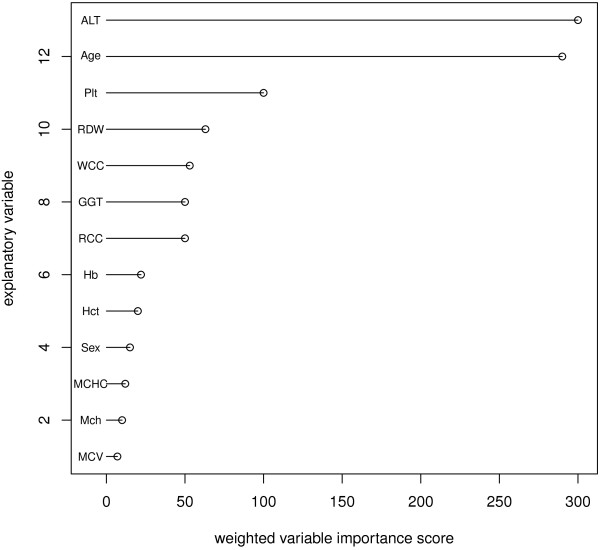
**Weighted importance for leading explanatory variables strongly linked to a positive HBSA immunoassay result.** Variable importance was calculated as the number of times a variable appeared in testing phase decision trees, Depth in decision tree weights indicates predictor variables at the top of the tree with the highest importance, with lower nodes contributing a lower weighting based on a lesser hierarchy importance.

To augment the prediction capacity of decision trees, decision tree ensembles can be constructed with the average of the multiple tree results used for accuracy prediction. Another advantage of this approach is that the relative importance of each explanatory variable used in the model can be estimated due to the average frequency of its appearance across the multiple trees comprising the ensemble (Figure 2).

For the basic multiple approach (Table 3a), the accuracy rates achieved for the prediction of positive HBSA were approximately 60%, which is lower than the rates achieved using a single tree with matched single pre-processing (Table 2). The best result overall was found for positive HepC (63.45%) using scaled data, but this results was < 1.0% different from the other positive HepC basic multiple results (for Raw, Log and Scale-Log). There was no need to scale and/or log the data prior to predicting positive HBSA. The Raw result (62.2%) was 2.4% superior to the various scaling and log pre-processing. For the basic multiple method, prior data scaling and/or log transformation had a negligible effect on prediction accuracy over that found for non-transformed raw data. For the prediction of negative HBSA or HepC, basic single and bootstrap single methods for single decision trees were vastly superior compared to basic multiple ensemble decision trees.

**Table 3:** Specificity and sensitivity (%) of HBV and HCV immunoassay outcome prediction after decision tree ensemble analyses

**(a) Measure**	**Raw**	**Scale**	**Log**	**Scale-log**
HBSA specificity	53.91	54.46	54.41	54.41
HBSA sensitivity	62.22	59.82	59.82	59.82
HepC specificity	57.75	57.65	57.77	57.66
HepC sensitivity	63.19	63.45	63.08	63.31
**(b) **Measure	Raw	Scale	Log	Scale-log
HBSA specificity	68.57	68.82	68.80	68.57
HBSA sensitivity	46.83	46.91	46.83	46.83
HepC specificity	58.87	58.91	58.88	58.87
HepC sensitivity	63.40	63.34	63.34	63.37
**(c) **Measure	Raw	Scale	Log	Scale-log
HBSA specificity	54.45	54.59	45.74	45.74
HBSA sensitivity	61.43	61.43	70.20	70.20
HepC specificity	35.04	34.87	36.90	36.88
HepC sensitivity	80.37	80.84	76.53	76.53

Methods employed were (a) basic multiple, (b) majority multiple and (c) clear negative analyses (see Methods). Prior to accuracy analysis, explanatory variables were subject to one of four pre-processing methods: none (raw), scaling, logging and scale-logging. Scaling sets the range of each explanatory variable to a common range of 0 – 100. Logging uses natural logarithm transformation. Scale-logging uses a common range of 0 – 100 then takes the natural logarithm.

Majority multiple pre-processing (Table 3b) found that the prediction of a negative virus infection result was superior for HBSA compared to HepC. For negative HBSA and HepC, scale and/or log transformation did not improve the performance of this prediction model, and again basic single and bootstrap single decision tree methods were superior (Table 2). For the prediction of positive HBSA or HepC results, prior scaling and/or log transformation did not improve percent accuracy beyond the results for raw, non-preprocessed data. However, this method did improve the results of single decision tree basic single and bootstrap single methods (Table 2). For positive HepC prediction, ensemble trees produced from non-preprocessed data (raw), scale, log and scale-log pre-processing methods produced similar prediction accuracy rates as a single tree, matched single method.

Finally, the clear negative method produced the best prediction accuracy for positive HBSA (70.2%) and HepC (80.84%) for decision tree ensembles (Table 3c). These results were also superior to matched single pre-processing for single decision trees (Table 2). For HBSA prediction from routine pathology data by a single decision tree, log or scale-log transformation were the best methods, while for HepC positive data scaling produced the best results, but only marginally higher compared to the raw (non-transformed) data (Table 3c). For negative HepC predictions by this method, with or without prior data transformation or processing, predictions were poor at 35 – 37%. Likewise, negative HBSA prediction was also poor with accuracy rates of 54.5% for raw or scale methods and 45.7% for log alone or scale-log methods for decision tree ensembles. The basic single decision tree (Table 2) was clearly the best methods for negative HBSA and HepC prediction.

Across the 12 experiments using ensemble classifiers, the best mean accuracy rate was obtained for positive HepC, using the clear negative method on scaled data. The results of the analysis of variance of the mean accuracy rates further explore the interactions of importance to these results (Table 4).

**Table 4:** Analysis of variance of mean accuracy rates for a four-factor experiment

**Source**	**SS**	**df**	**MS**	**F**	**p**
Method	28.015	2	14.008	0.488	0.620
Pre-processing	0.967	3	0.322	0.011	0.998
Virus	44.815	1	44.815	1.560	0.224
Outcome	927.169	1	927.169	32.279	0.000 (*)
Method.Outcome	2909.082	2	1454.541	50.640	0.000 (*)
Method.Pre-processing	0.863	6	0.144	0.005	1.000
Method.Virus	42.649	2	21.324	0.742	0.487
Pre-processing.Outcome	8.436	3	2.812	0.098	0.960
Virus.Outcome	922.604	1	922.604	32.120	0.000 (*)
Pre-processing.Virus	0.301	2	0.100	0.003	1.000

The experiment examines interactions affecting the prediction of HBSA and HepC immunoassay outcome.

(*) = Significant at 0.001 level.

Method = basic single, basic multiple, majority multiple or clear negative.

Pre-processing = none, log, scale, or scale-log.

Virus = Hepatitis B or Hepatitis C.

Outcome = positive or negative.

Method.Outcome = the interaction between method and outcome; other interactions between pairs of variables to be interpreted similarly.

### Analysis of variance

**Figure 3:**
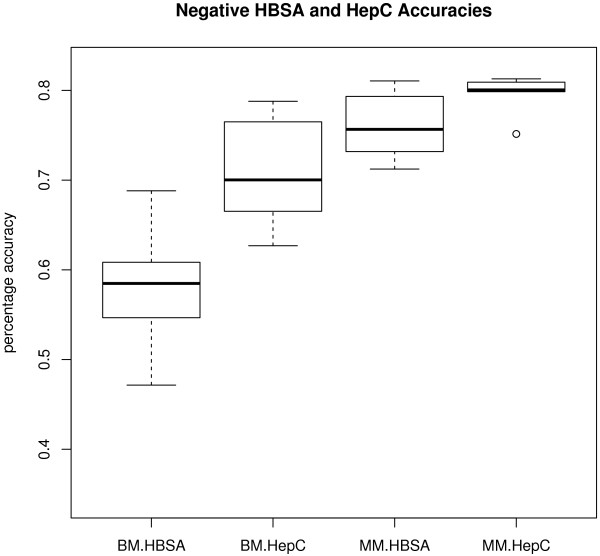
**Sensitivity and specificity for the best method of data pre-processing - Negative viral infection.** Sensitivity and specificity rates are shown for the scale method of pre-processing associated with negative hepatitis B virus (HBV) or hepatitis C virus (HCV) infection, including the two outcomes (positive or negative prediction). BM = Basic multiple approach. MM = majority multiple approach.

**Figure 4:**
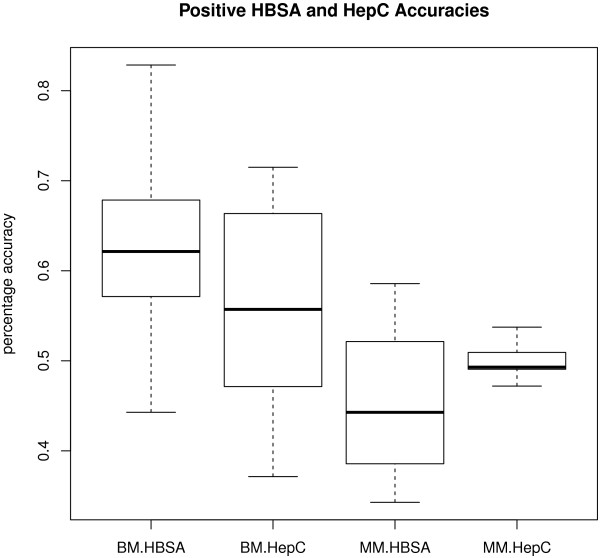
**Sensitivity and specificity for the best method of data pre-processing - Positive viral infection.** Sensitivity and specificity rates are shown for the scale method of pre-processing associated with positive hepatitis B virus (HBV) or hepatitis C virus (HCV) infection, including the two outcomes (positive or negative prediction). BM = Basic multiple approach. MM = majority multiple approach.

The analysis of variance (Table 4) shows that accuracy rate depends on outcome (F = 32.279, df = 1 and 23, p = 0.000). Positive cases have a higher accuracy rate on average than negative. There is also a significant interaction between method (e.g. basic multiple) and outcome (e.g. predicted positive: F = 50.640, df = 2 and 23, p = 0.000). Majority multiple does better on average at predicting negatives, whereas the other two methods perform better on average at predicting positives. The other significant interaction is between virus type (HBV or HCV) and outcome (F = 32.120, df = 1 and 23, p = 0.000). While HepC positive status leads to higher accuracy rates on average, the reverse is true for HBSA. Since basic multiple and majority multiple methods produce 72 and 36 accuracy rates respectively, our results include boxplots of the range of accuracy rates for those methods across the two viruses and two outcomes (Figures 3, 4). Figures 3 and 4 summarise results only for the scale method of pre-processing, since that was found to be the most successful experiment (see above). The other pre-processing results are similar, which are confirmed by the non-significant effect of pre-processing in the analysis of variance in Table 4.

The location of each mean accuracy rate shown in the centre of the boxplot is confirmed by the figures in the “scale” column of Table 3. The spread of the accuracy rates is slightly larger for positive outcomes compared to negative outcomes. This strengthens the significant effect of outcome on accuracy rate found in the analysis of variance above. There is not much difference in the accuracy rate distributions for the two viruses, which also strengthens the non-significant effect of virus on accuracy rate found in the analysis of variance (Table 4).

## Discussion

Infection by Hepatitis B virus (HBV) or Hepatitis C virus (HCV) are significant agents of acute and chronic hepatitis world-wide, and leading causes of liver cancer and cirrhosis. Prevalence rates can vary widely between different countries; for example, HBV carrier prevalence within Europe ranges from 0.1 to 8.0% and HCV from 0.1 to 6.0% [8]. The health impact of HBV worldwide is substantial with 2 billion cases of infection, 360 million cases of chronic infection and 600,000 deaths each year associated with liver carcinoma or other HBV-induced liver disease [9]. Based on WHO estimates from 1999, worldwide HCV prevalence was around 3.0%, with approximately 170 million people affected by HCV disease. Due to prolonged disease latency post HCV infection, prevalence rates are difficult to calculate, so the quoted rates may be underestimated [10].

Primary diagnosis of HBV or HCV, and subsequent monitoring of infection, relies significantly on immunoassay techniques available via pathology departments to detect hepatitis B virus surface antigen (HBSA), or patient anti-HCV antibodies (HepC) associated with previous infection. Within the suite of immunoassay markers available for HBV detection, HBSA was chosen since it is a common HBV screening test and is elevated relatively soon after infection (Table 1). For all analyses, HBSA or HepC were used as the respective response variables in the single and ensemble decision tree methods. The explanatory variables used for all analyses comprised a range of other routine pathology tests run simultaneously with the HBV or HCV immunoassay on the same serum samples. These additional tests reflect a number of physiological functions that are potentially perturbed by infection and illness, including liver function, kidney function, presence of anaemia and infection or allergy (Table 1). Judgments based on the linear reference ranges decided by the laboratory for individual assay results assist in both primary diagnosis and subsequent monitoring of disease or infection, if present.

Given the extensive range of biochemical, cellular and physiological data available associated with HBV or HCV immunoassay via simultaneously collected routine pathology laboratory results, a pattern recognition approach beckons to reveal data patterns that reflect both the presence of HBV/HCV infection, as well as infection persistence and severity (via follow up data). To address this opportunity, single decision trees and tree ensembles were employed. A tree ensemble consists of several individually trained trees that are jointly used to solve a problem. Given the over-representation of negative cases in both the HBV and HCV data, tree ensembles can give a significant improvement in prediction accuracy over a single classifier. Generally, constructing ensembles consists of two phases, a training phase and a combining phase [11,12].

In the training phase, several techniques to cope with the imbalanced nature of the data were explored. One popular method for balancing a training set is bootstrapping [13]. This technique generates a training set using random drawing (with replacement) from the original training set. Consequently, in every new training set there are data points that appear more than once while others do not appear at all. Bootstrapping is an effective technique for improving a classifier with poor performance, especially where a classifier has been presented with a small training sample set or training set with misleading data points. A second method involves downsizing the large class either at random or at “focused” random [14,15]. Training sets were produced using a subset of the negative individuals, as there are many more negatives than positives in both the HBV and HCV data sets.

In the combining phase, we have chosen to use a majority voting strategy to combine predictions of the component classifiers. In majority voting each component classifier votes for a category, and the category with the majority of votes defines the ensemble category. The best approach for negative HBSA and HepC data accuracy was the “basic single” method (see Table 2) due to the size of these datasets.

For smaller datasets, as found for both HBSA and HepC positive cohorts, other methods were required to achieve high predictive accuracy based on associated routine pathology data (Table 1). Furthermore, the “clear negative” method, which used other pathology data (i.e. ALT liver enzyme) to give the most certain true negative cohort, was very effective. For this method, patient data with HBSA < 0.01 and ALT < 55 U/L were considered to be “clear negative” for HBV. We also considered patient data with HepC ≤ 0.03 as “clear negative” for HCV. Such combining of diverse pathology data to increase the probability of a correct true negative or true positive detection is particularly crucial in the context of blood transfusion, where the accidental transmission of infectious agents must be avoided [16].

## Conclusions

This study examined the effect of data characteristics on decision trees used to predict HBV or HCV infection status, as detected by specific immunoassay. Improved understanding of the behaviour of such techniques will lead to the better definition of patient groups that display different data patterns associated with HBV or HCV infection, and hence demonstrate a different physiological response as defined by biochemical and cellular responses to infection, determined by routine pathology blood tests. Once rules are determined via data mining, patient profiles can be designed that will guide molecular genetic studies on the biological basis of disease resistance or susceptibility, with the shorter term benefit of enhancing the laboratory diagnosis and monitoring of hepatitis virus infection through combined data rules, particularly for data sets with few positive cases. This study focused on interactions between aspects of the data and its pre-processing that allow decision trees to generate effective rules, which model hepatitis virus infection, derived from routine blood test data that assesses liver and kidney function, as well as a range of markers that explore red and white blood cell function.

## Methods

### Software

We implemented the analysis using the RPART algorithm in R [17]. Post decision tree construction (single and ensemble) prediction accuracy rates were measured as in [18] by sensitivity and specificity. Sensitivity and specificity are defined as follows:

$$\begin{array}{rl} \mathrm{sensitivity} & =\;\frac{\mathrm{TP}}{\left(\mathrm{TP}+\mathrm{FN}\right)}\;\mathrm{and}\\ \mathrm{specificity} & =\;\frac{\mathrm{TN}}{\left(\mathrm{TN}+\mathrm{FP}\right)} \end{array}$$

where the true positive (TP) is the number of correctly diagnosed HBSA or HepC positive cases; false negative (FN) is the number of HBSA or HepC positive that the model is unable to diagnose; true negative (TN) is the number of correctly diagnosed HBSA or HepC negative successfully diagnosed by the model; and false positive (FP) is the number of HBSA or HepC negative that the model is unable to diagnose.

### Data set

The data set employed in this study originally comprised 18625 individual cases (1 individual patient case per row) of hepatitis virus testing over a decade from 1997 – 2007. Data was provided by ACT Pathology, The Canberra Hospital (TCH), Australia. Patient identifiers were removed by TCH staff prior to data access, with only a laboratory ID numbers provided for the study. After data cleaning that included the removal of rows with missing values, 10378 rows of complete data were compiled for HBSA, with 8801 complete data rows available for HepC. Only cleaned and complete data sets were used in the experiments described herein. Of the final data set, 212 rows were HBSA positive, and 641 rows positive for HepC. Therefore, the majority of the data were negative for either HBV or HCV, stimulating the analyses described here to derive methods to increase prediction accuracy for an unbalanced data set. HBSA was classified as positive at ≥ 1.6 immunoassay units (IU) and HepC ≥ 0.6 IU for a positive classification. All other HBSA and HepC results below this assay cut-off were classified as negative (M. de Souza, ACT Pathology, pers. comm.).

The study was divided into two phases to assess the impact of pre-processing efficacy. The first phase compared three pre-processing techniques before testing accuracy for single decision trees. Phase two comprised ensembles of 36 or 72 decision trees with pre-analysis scaling of the data before an assessment of prediction accuracy.

### Ethics

For access to de-identified patient data, this study had human ethics approval granted by the Human Research Ethics Committee at The University of Canberra (protocol 07/24), The Australian National University Human Ethics Committee (2012/349) and the ACT Health Human Research Ethics Committee (ETHLR.11.016).

### Phase 1 – single decision tree analysis

Prior to running the single decision trees and assessing prediction accuracy, four common data pre-processing techniques were employed [19]. The four pre-processing techniques used were: no pre-processing (Raw), scaling 1 – 100 (Scale), a natural logarithm scale (Log) and scale-logging (Scale-Log), a combination of the previous two methods. Scaling sets the range of each explanatory variable to a common range of 0 – 100. Logging uses the natural logarithm (ln) transformation. Scale-logging uses a common range of 0 – 100 then takes the natural logarithm. Note also that assignment of positive or negative to data (based on HBSA or HepC value) occurs before scaling.

After data pre-processing, three data set selection methods were used, as follows;

Basic Single: For both HBSA (n = 10378) and HepC (n = 8801), two-thirds of the data were randomly selected for training with the other third of data reserved for testing [19]. The single tree obtained from the training set was applied to the testing set, and the accuracy rate computed.

Bootstrap Single: Pre-processing was identical to the basic single approach, but in addition used the bootstrap technique in order to increase the number of positive cases in the training data to match the same number of negative cases (in the training phase). The bootstrapped training data is then used to construct a tree classification for the response variables HBSA and HepC, which were compared for accuracy to the one-third testing data.

Matched Single: As an alternative to bootstrapping, the same number of negative cases as the available number of positive cases were used to train the data, with the negative cases selected at random from the whole data set. This training data was then used to construct a tree classification for the response variables HBSA and HepC, as summarized above.

### Phase 2 – decision tree ensembles

As well as the single decision tree methods above, it is also possible to divide up the abundant negative cases into multiple sets (see [14]) and thereby produce multiple decision trees. Three methods for carrying out this division were studied: a description of each one follows.

Basic Multiple: Positive HBSA data was randomly divided into two parts comprising of 2/3 training data (141 cases) and 1/3 testing data (71 cases). Cases with negative HBSA (10167 cases) were selected, and divided into 72 random subsets (i.e. 10167/141). The 141 cases with positive HBSA were combined with each of the above 72 HBSA negative subsets. The above 72 subsets were applied one at a time to construct a classification tree for the response variables, and each of the original 72 trees applied to the remaining data, which had not been used in construction of that individual tree, and compute the accuracy rate for each subsequent tree ensemble.

Majority Multiple: This method is very similar to the basic multiple method. However, this time we created 36 subsets for training where each subset had 282 (i.e., 141 × 2) cases with half cases negative and the other half of cases with positive HBSA. Furthermore, we computed the accuracy rate for each tree (using the same test dataset) based on majority voting from all trees. The result of a majority vote is the decision of at least > 50% of the trees in the ensemble.

Clear Negative: For this method we first select the cases that are “clearly negative” as judged by pathology data reference ranges for HBSA or HepC immunoassay and ALT (Table 1). “Clear negativity” is defined as HBSA < 0.01 IU, HepC < 0.03 IU and ALT < 55U/L. We then combined them with 2/3 of the positive cases (71 for HBSA or 214 for HepC) to construct the training set. The remaining data are used for testing. In other words, we have only one set for training and the remaining data for testing, unlike the other two methods that had multiple training sets.

### Phase 3 – analysis of variance

The final phase of the study uses an analysis of variance [20] in order to identify the amount of variation in mean accuracy rate attributable to four factors: method, data pre-processing, outcome and virus type (HBV or HCV). There are three methods (basic multiple, majority multiple and clear negative). There are four pre-processing techniques (none, scale, log, and scale-log). There are two outcomes (predicted positive and predicted negative). There are two viruses, Hepatitis B virus (HBV -measured by the immunoassay marker HBSA) and Hepatitis C virus (HCV – measured by the immunoassay marker HepC: Table 1). The interaction between pairs of these factors was also modelled, to see if there were settings of one factor that caused the accuracy rates to behave differently depending on the setting of another factor.

## Competing interests

The authors declare that they have no competing interests.

## Authors’ contributions

AMR supervised the decision tree construction and conducted the analysis of variance. BAL negotiated access to pathology data, conducted the interpretation of the decision trees, and researched the biomedical importance of the results. Both authors contributed equally to the writing of the paper. Both authors read and approved the final manuscript.
